# the relationship between childhood sexual abuse and the developement of schizophrenia, ptsd and substance abuse among men through life

**DOI:** 10.1192/j.eurpsy.2025.2229

**Published:** 2025-08-26

**Authors:** M. Kupchik, S. Mustafa, N. Sinai

**Affiliations:** 1 MERHAVIM MENTAL HEALTH CENTER BEER YAAKOV-NESS ZIONA, NESS ZIONA, Israel

## Abstract

**Introduction:**

**Background and aims:** There is a lack of research regarding the topic of childhood sexual abuse trauma on men population. Childhood experiences and traumas can have a great impact on various life domains, including mental state and addictions.

This study examines the impact of childhood sexual abuse on men diagnosed with schizophrenia – regarding the age of the disease’s appearance, the appearance of additional PTSD diagnosis and substance use throughout life.

**Objectives:**

This study can contribute to the development of more effective treatment and prevention strategies for men who have experienced childhood sexual abuse and are diagnosed with schizophrenia.

**Methods:**

Participants: 60 Male patients hospitalized in the closed wards for men, aged 18-50 at ‘Merhavim’ - Mental Health Center.

Data Collection: A one-time meeting with the research team, which included completing several questionnaires regarding their current state and childhood experiences.

**Results:**

The influence of childhood sexual abuse was significant in all subjects we examined. Patients of suffered from sexual abuse in their childhood were more likely to develop PTSD or develop schizophrenia at a much younger age. Also, they were more likely to suffer from substance abuse throughout their life.

**Conclusions:**

- Childhood sexual abuse has a significant impact on the onset of schizophrenia symptoms (age of first hospitalization), PTSD symptoms and substance abuse throughout life.

- Traumatic childhood experiences have a significant impact on mental symptoms that appear in adulthood as well as on various behaviors throughout life.

**Disclosure of Interest:**

None Declared

